# COVID-TRACK: world and USA SARS-COV-2 testing and COVID-19 tracking

**DOI:** 10.1186/s13040-021-00233-2

**Published:** 2021-01-20

**Authors:** Ye Emma Zohner, Jeffrey S. Morris

**Affiliations:** 1 Department of Statistics, Rice University, 6100 Main Street, Houston, TX 77005 USA; 2 Department of Biostatistics, Epidemiology and Informatics, Perelman School of Medicine, University of Pennsylvania, 600 Blockley Hall, 423 Guardian Drive, Philadelphia, PA 19104 USA

**Keywords:** COVID-19, SARS-COV-2, Web-application, COVID data science, COVID tracking

## Abstract

**Background:**

The COVID-19 pandemic has caused major health and socio-economic disruptions worldwide. Accurate investigation of emerging data is crucial to inform policy makers as they construct viral mitigation strategies. Complications such as variable testing rates and time lags in counting cases, hospitalizations and deaths make it challenging to accurately track and identify true infectious surges from available data, and requires a multi-modal approach that simultaneously considers testing, incidence, hospitalizations, and deaths. Although many websites and applications report a subset of these data, none of them provide graphical displays capable of comparing different states or countries on all these measures as well as various useful quantities derived from them. Here we introduce a freely available dynamic representation tool, COVID-TRACK, that allows the user to simultaneously assess time trends in these measures and compare various states or countries, equipping them with a tool to investigate the potential effects of the different mitigation strategies and timelines used by various jurisdictions.

**Findings:**

COVID-TRACK is a Python based web-application that provides a platform for tracking testing, incidence, hospitalizations, and deaths related to COVID-19 along with various derived quantities. Our application makes the comparison across states in the USA and countries in the world easy to explore, with useful transformation options including per capita, log scale, and/or moving averages. We illustrate its use by assessing various viral trends in the USA and Europe.

**Conclusion:**

The COVID-TRACK web-application is a user-friendly analytical tool to compare data and trends related to the COVID-19 pandemic across areas in the United States and worldwide. Our tracking tool provides a unique platform where trends can be monitored across geographical areas in the coming months to watch how the pandemic waxes and wanes over time at different locations around the USA and the globe.

## Background

Coronavirus-2019 (COVID-19) is an infectious disease caused by a newly discovered coronavirus, SARS-CoV-2. The novel coronavirus was declared a Public Health Emergency of International Concern on January 30, 2020 and declared a pandemic on March 11, 2020 by the World Health Organization (WHO) [1]. Most people infected with the COVID-19 virus experience mild to moderate respiratory illness and recover without requiring special treatment [2]. However, a subset suffers severe symptoms and is at risk of long-term hospitalization or death, especially older people and those with underlying medical problems such as cardiovascular disease, obesity, diabetes, chronic respiratory disease, and cancer [3]. It is thought to spread mainly from person to person, mainly through respiratory or aerosol droplets produced when an infected person coughs or sneezes, or sometimes through touching infected surfaces [4]. Infections can occur when enough volume of the virus enters the respiratory system through the mouth or nose through these respiratory or aerosol droplets, or by touching one’s face after contracting sufficient volume of viral particles from touching contaminated surfaces [1]. The median incubation period of COVID-19 is estimated at 5.1 days and most cases who develop symptoms will do so within 14 days [5].

As of June 2020, there are no vaccines or proven treatments for COVID-19, although numerous vaccines are under rapid development [6] and many ongoing clinical trials of numerous treatments [7]. The COVID-19 pandemic has caused major socioeconomic disruptions in the United States and worldwide. As SARS-CoV-2 has spread throughout the world, different places have encountered the pandemic with varying degrees of severity, and different municipalities have approached testing and viral mitigation in different ways, with some countries quickly ramping up testing and others lagging, with some quickly instituting social distancing measures and stay-at-home orders, and others using less aggressive strategies, and different places relaxing these social distancing measures at different rates [8, 9]. Upticks and surges of SARS-CoV-2 have resulted after reopening of businesses and stay-at-home orders in some USA states, particularly in the south and west, while other states have showed continuing decline or stabilization of cases after reopening. This diversity in response makes the tracking of data and study of outcomes crucial for documenting and learning from the COVID-19 outbreak.

To understand how the pandemic has unfolded and follow how it changes over time, it is important to be able to compare different countries and different states to identify potential emerging hotspots and assess potential effects of different mitigation strategies. These comparisons must necessarily include simultaneous assessments of incidence, mortality, testing, and hospitalizations given that these different measures each contain complementary information about the disease and their interpretations are interrelated. For example, incidence is directly affected by testing rate. To assess whether increasing incidence is evidence of a surge in infections one must check the corresponding increase in testing. Test positivity rate seems to be a useful derived measure, tending to decrease as testing practices change to include more mild or moderate cases and tending to increase during a surge in infections [10]. Similarly, death rate per case, if higher, could indicate an overwhelmed hospital system or older demographic, or more likely could simply be an indication that local testing practices are only capturing severe, not moderate or mild cases.

Finding accurate data for tracking facets of the COVID-19 pandemic is challenging. Different jurisdictions record and report data differently, and it is challenging to compile this complex data in a timely manner. Two sources have worked to provide accurate and reliable data: The COVID Tracking Project at https://covidtracking.com for United States data, and Our World in Data at https://covid.ourworldindata.org for international data. The COVID Tracking project has aggregated and curated data on SARS-CoV-2 testing across states in the United States, while documenting specific recording practices of individual states, e.g. whether duplicate tests on same person are reported, and also recording cases, deaths, and hospitalizations where available. The Our World In Data site aggregates testing, incidence, and deaths from all around the world. These rich data sources are the most complete assembled, but unfortunately their websites do not have complete plotting capabilities to accommodate detailed comparisons of viral characteristics of different states or countries. Covidtracking.com does not provide direct plotting routines, and ourworldindata.org allows plotting of deaths, cases, and tests, but has some limitations, e.g. not computing or plotting derived quantities like test positivity rate or deaths per case.

There are currently many websites and web-applications for tracking data related to COVID-19. The STAT Covid-19 Worldwide dashboard plots worldwide and US cases, hospitalizations, and deaths. The plots show number of days since first case as well as a heatmap of daily changes in new cases and deaths. However, they do not provide testing information, per capita values, moving average or derived quantities such as proportion of deaths per positive test [11]. The John Hopkins University & Medicine COVID-19 Dashboard provides world and US maps of cases, incidence rate, fatality rate and testing rate. The dashboard also shows a line chart of confirmed cases and a histogram of deaths overtime. For US data, the plots can be seen by county. Although there is a page for critical trends analysis, one cannot compare trend lines or different jurisdictions on the dashboard, and view trends per capita or on a log scale [12]. The Centers for Disease Control and Prevention (CDC) COVID Data Tracker shows a map and a bar chart of US cases by state. The user can hover on the figure for death numbers, cases in last 7 days and cases per 100 k. The CDC dashboard also allows a comparison of state trends for cases and deaths. The comparison can be done per capita on a linear or logarithmic scale. However, the site limits data and comparisons to the US [13]. Wordometer shows a tabular representation, bar charts and line charts for worldwide and US cases, deaths and number recovered at the country and US state level. For several US states, county level data is given. One can view plots for single jurisdictions but there is no option to compare trends for multiple jurisdictions [14]. CNN sources data from John Hopkins University [15] and provides a COVID-19 tracking dashboard that shows maps and tables of case numbers, case numbers per capita, deaths, and deaths per capita by US state and county. The CNN dashboard also provides case growth rates in the US and by state, as well as bar charts and trend line for daily new cases. The visualizations include histograms of daily new cases by states, with smoothing lines. Non-US data is not shown. The New York Times provides world and US map of cases, cases per 100 k, deaths, deaths per 100 k, and a growth rate heatmap. There are single countries/states trend plots where cases are increasing, mostly the same and decreasing. However, the trend plots are static and do not allow one to toggle between raw numbers and per capita counts [16]. Microsoft Bing’s COVID-19 Tracker shows world and US state and county maps, bar charts and line charts of active, recovered, and fatal cases, for cumulative and daily. The application gives an animated map of case progression overtime. A number of plots including line charts, histograms, and tree maps display counts of confirmed, active, recovered and fatal counts on a linear or logarithmic scale. One can compare each country to the top 10 countries with the highest number of cases. There is no option to compare several countries over than the top 10. In addition, since one plot gives the option to view different epidemiology variables with a drop down (confirmed, active, recovered, and fatal), one cannot view all four variables at once [17]. CoronaTracker reports world and US data with line charts and bar charts for cases, recovered, deaths as well as fatality and recovery rates. Each country can be viewed individually [18]. Outbreak.info shares maps, tables and histograms of testing, cases, hospitalization, and deaths, comparing countries and US regions. The dashboards offer choices among many epidemiology variables and give an option to view counts on a linear or log scale. Histograms and corresponding smoothing plots can be viewed side by side for different jurisdictions [19].

Although all these websites present useful data and visualization about the virus and the disease, they do not particularly focus on analyzing testing, incident, hospitalization, and death trends across jurisdictions. As a result, we set out to develop a dynamic representation tool to provide a readily accessible visual representation of the daily evolution of testing, incidence, hospitalization, and deaths for US states or countries, providing a flexible set of transformations and derived quantities to allow users to explore many different aspects of the pandemic and compare various states or countries. The web-application provides:

1. Different figures for testing, incidence, hospitalizations, and death which provides a holistic view of the situation: Incidence data is impossible to interpret relative to growth unless we understand the testing rate. In addition, not all cases are equally severe in all places. Hospitalization and death data convey information about severity of cases in each area.
2. Comparisons between USA states or countries worldwide: different jurisdictions have different levels of outbreak and implemented different testing and social distancing policies at different timelines. Significant insights can be obtained by the concurrent analysis of trends across several states or countries.
3. Cumulative, per capita or incremental results: Testing, cases, hospitalizations or deaths can be viewed cumulatively or per day, depending on the need, and raw counts or per capita counts can be used to allow adjustment for heterogeneous population sizes.
4. Raw data, 3-day moving average, 7-day moving average: Some municipalities report test, cases, deaths unevenly overtime that lead to visual artifacts in the raw data that obscure time trends. Moving averages smooth out the discrepancies in reporting intervals.
5. Raw data or log scale: Raw or log scale data can provide different views of the outbreak, e.g. assessing whether growth trends are linear or exponential.
6. Transformations and rates: Various derived quantities are useful to track to assess local outbreak characteristics, including test positivity rate, deaths per case, and hospitalizations per case.

To our knowledge, no website or web-application plots all of these data and provides all of these options.

We have developed COVID-TRACK and make it freely available to scientists and the community at large. This provides a platform to:

1. Aggregate and present daily SARS-CoV-2 testing, and COVID-19 incidence, hospitalization, and death as accurately as possible, in the United States and worldwide.
2. Compare states and countries in a way that allows users to gain a complete picture of how the pandemic experience has varied across locations and changed over time.
3. Identify emerging patterns and compare jurisdictions incorporating different mitigation strategies.

Our application is connected to the daily-updated data sources, and automatically updates with each day’s new data.

### Implementation

We developed the web-application in Python Dash. Dash is an Open Source Python library for creating reactive, analytical web-based applications. The data sources include the COVID Tracking Project and Our World in Data, as well as USA population and world population data. The web-application can be found at https://covid-track-app.herokuapp.com. In our implementation, the user can choose any number of states in United States, or number of countries in world, and plot the following information in 4 figures:

1. Tests conducted,
2. Positive tests,
3. Hospitalizations, and
4. Deaths

The user can plot the data (a) cumulatively, (b) daily. The cumulative and daily data can be plotted for a) raw values, b) per million resident or c) per unit. The “per unit” option plots various derived quantities or rates, including:

1. Proportion of tests per resident in the jurisdiction,
2. Proportion of positive tests per test taken,
3. Proportion of hospitalized per positive test, and
4. Proportion of deaths per positive test.

**Fig. 1:**
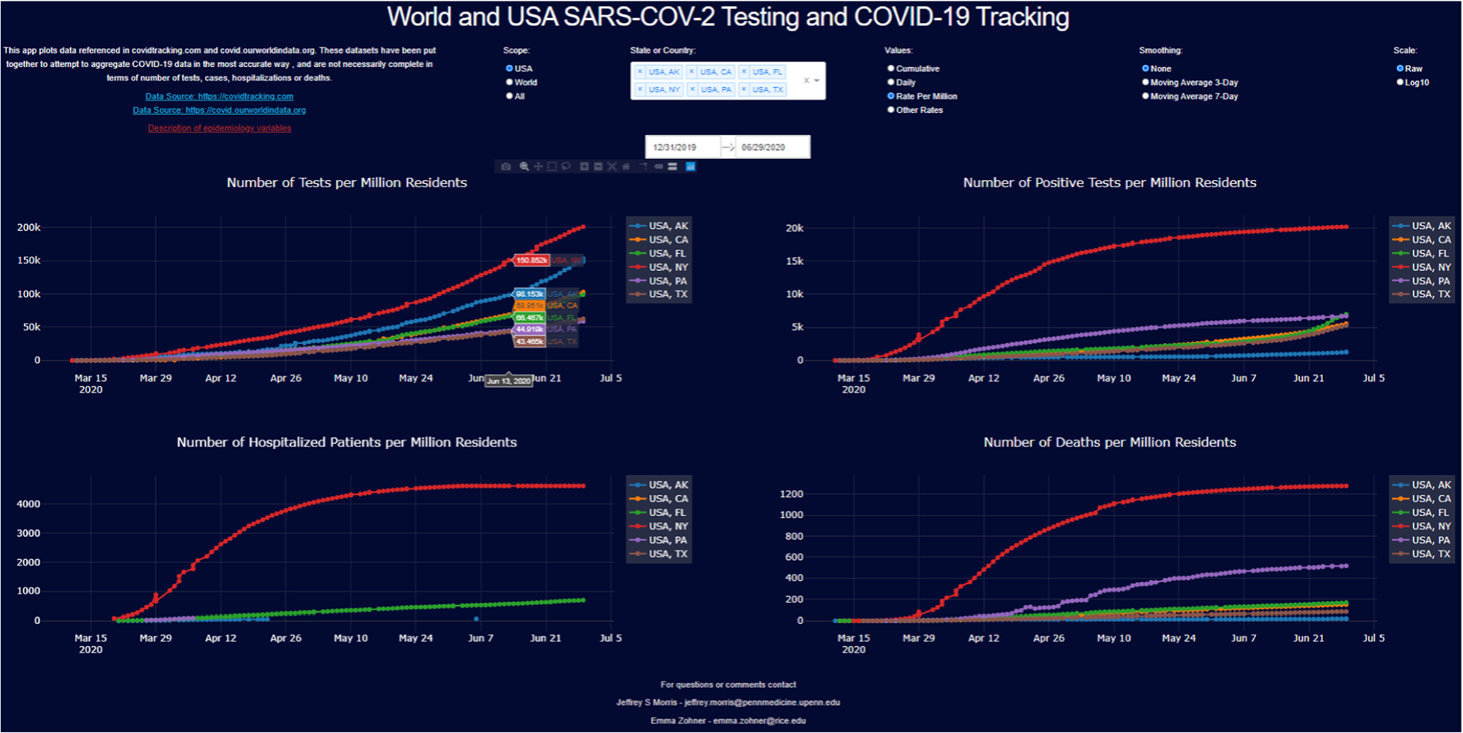
Web-app: user can select a scope, country or state, values (cumulative, incremental, per million or rates), aggregation (raw, 3-day, 7-day moving averages) and scale (raw, log base 10)

Additionally, the data can be plotted in raw numbers, or 3-day and 7-day moving averages. The user can select a raw scale or a log base 10 scale. To see actual numbers, the user can hover on a location in each plot (Fig. 1).

## Results and discussion

COVID-TRACK is useful for investigating how different countries or USA states have experienced the pandemic over time. This analysis demonstrates how much it is imperative to simultaneously consider testing, incidence, and deaths as well as testing positivity rate in order to get a complete, accurate picture of pandemic experience and make a fair comparison across countries or states with different testing practices, populations, or testing practices varying over time.

### Scandinavia and Europe

**Fig. 2:**
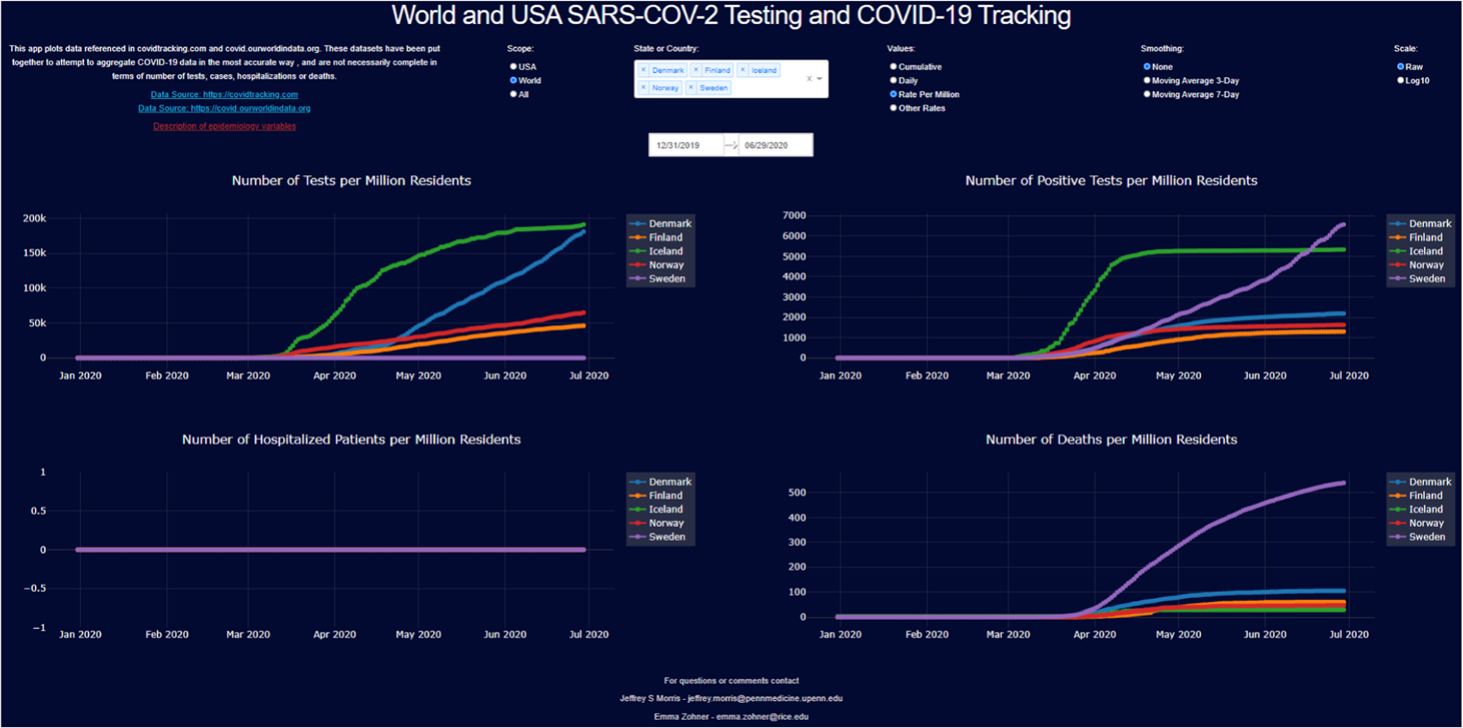
Scandinavia per million residents cumulative counts - top left: number of tests, top right: number of cases, bottom right: number of deaths

Sweden has drawn global attention because of its uniquely relaxed approach to the COVID-19 pandemic [20]. Contrary to other Scandinavian and European countries, Sweden did not impose strict stay-at-home restrictions, lockdowns, or close schools and restaurants, but instead left society open while providing some social distancing and mask-wearing recommendations. Comparing Sweden to its Scandinavian neighbors (Norway, Denmark, Finland, and Iceland), the per capita trends suggest that Sweden has noticeably higher per capital incidence than Denmark, Finland, or Norway, and has recently surpassed Iceland in per capita cases (Fig. 2). Looking at testing data, we see Iceland’s high incidence is from its extremely high per capita testing rate, among the highest in the world, which is also manifest in the extremely low test positivity rate. This indicates that testing is broadly done, and with exceptionally low deaths per positive test, suggesting Iceland’s cases include many with far less severe disease that is not fatal, and is the result of their aggressive population-level testing strategy. Not only is incidence high in Sweden, but testing also lags behind its neighbors, with the lowest per capita testing rate in Scandinavia, and by far the highest test positivity rate and deaths per positive test.

**Fig. 3:**
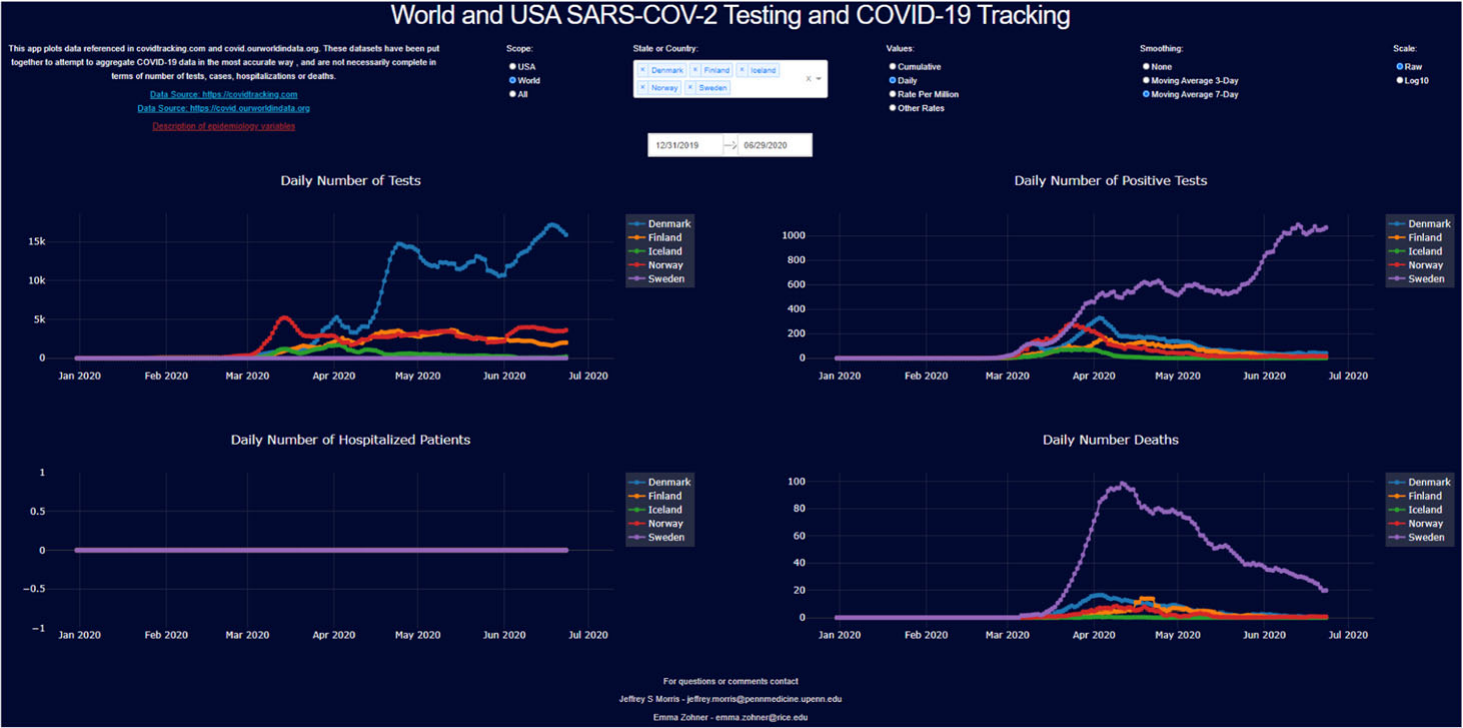
Scandinavia 7-day moving average of daily counts - top left: number of tests, top right: number of cases, bottom right: number of deaths

Moving from cumulative to daily results, cases are still increasing in Sweden, while the daily cases and deaths have been decreasing in the rest of Scandinavia (Fig. 3).

**Fig. 4:**
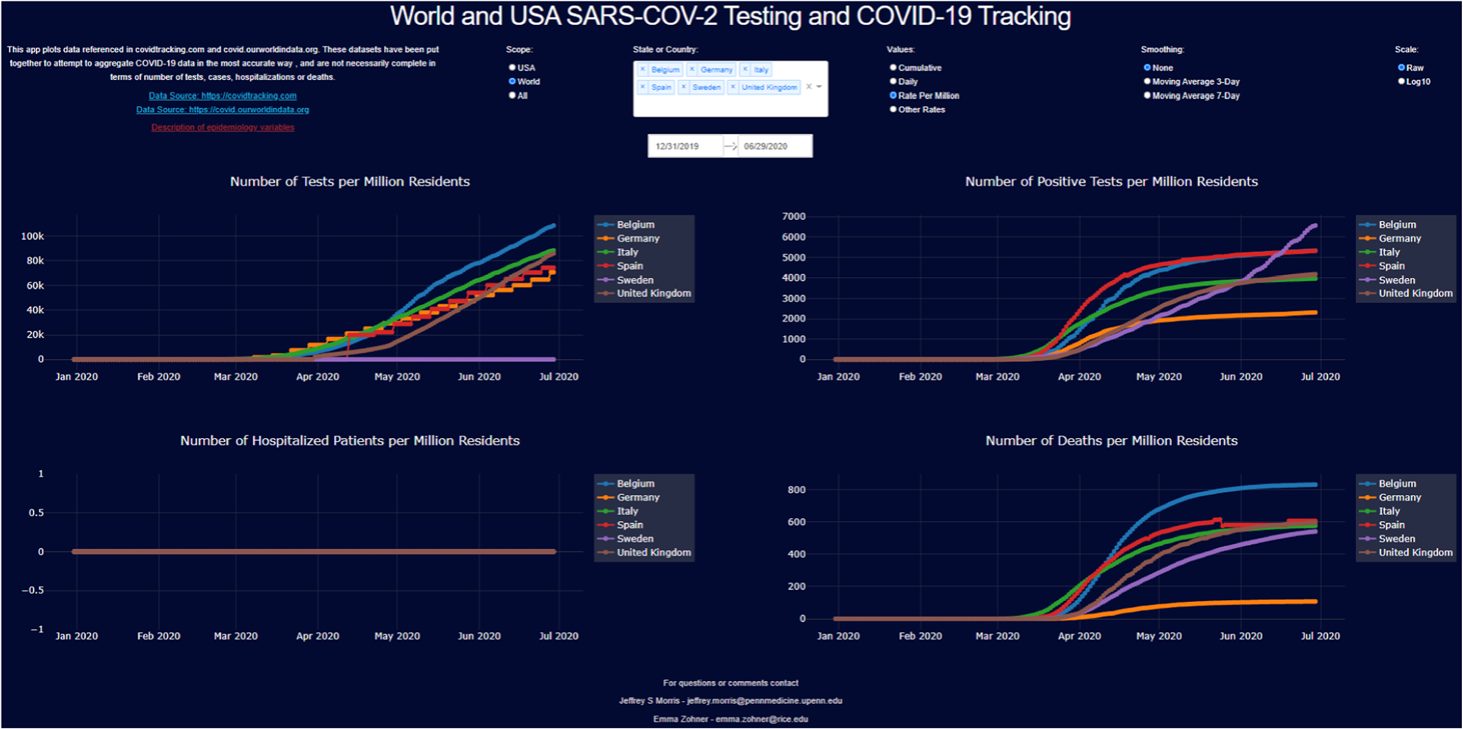
European countries per million residents cumulative counts - top left: number of tests, top right: number of cases, bottom right: number of deaths

The overall impact of the virus in Sweden was not the worst in Europe until recently, with other countries such as Belgium, Spain, and the United Kingdom experiencing high death rates. However, while other countries show flattening in incidence and death curves, Sweden still shows a steep slope. These data put the Swedish experience into perspective, and any attempt at explanation using only a subset of these data would miss an important part of the story (Fig. 4).

### Worldwide early measures

**Fig. 5:**
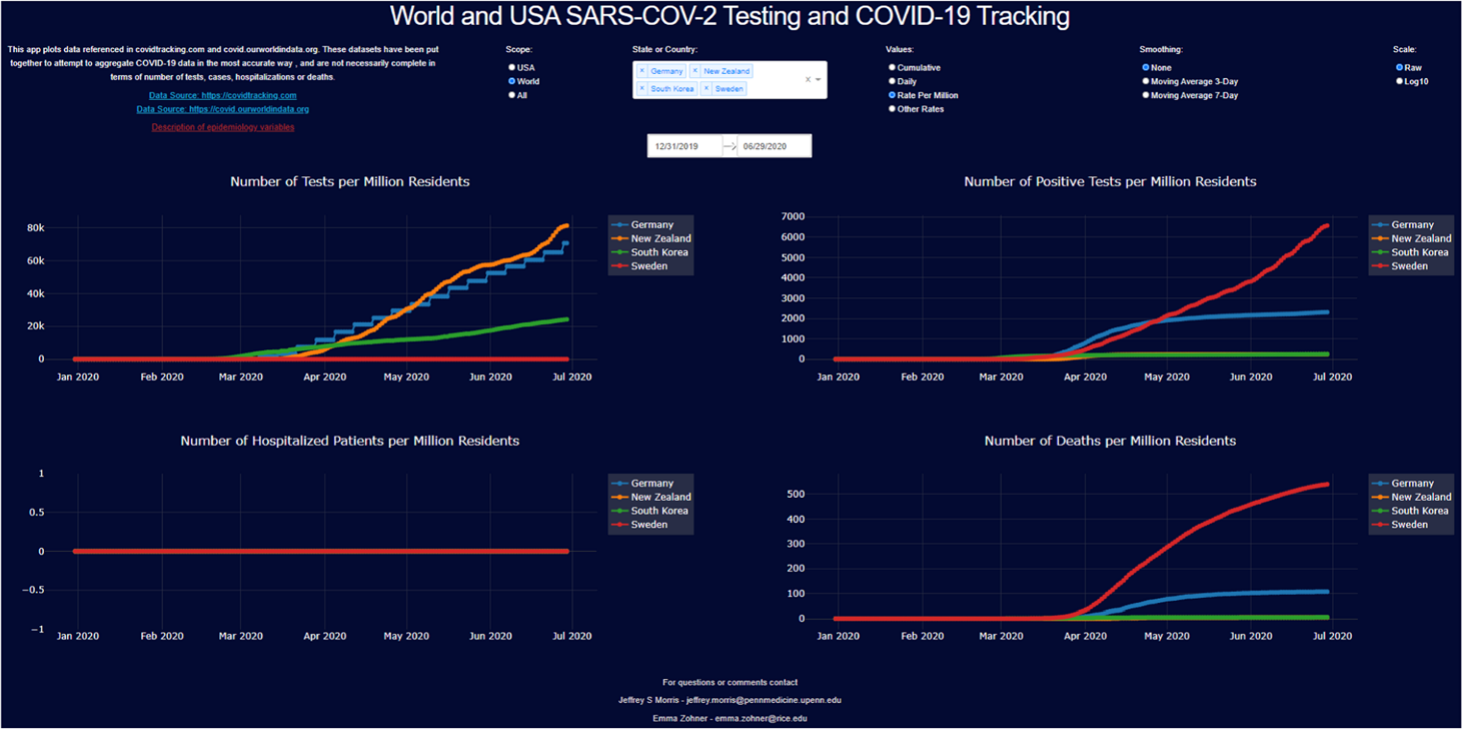
Early measure comparisons per million residents cumulative counts - top left: number of tests, top right: number of cases, bottom right: number of deaths

In contrast to Sweden and other countries, Germany, South Korea, New Zealand, and Canada implemented testing and tracing measures early [20]. In comparison, countries who took early measures seem to have fared considerably better in terms of per capita incidence and death (Fig. 5).

### United States of America

**Fig. 6:**
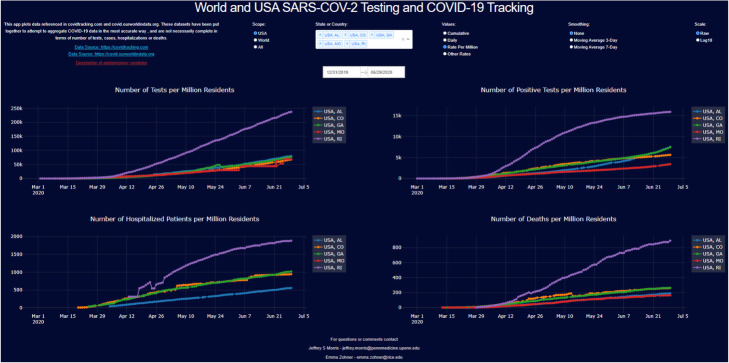
USA (AL, CO, GA, MO, RI) per million residents cumulative counts - top left: number of tests, top right: number of cases, bottom left: number of hospitalizations, bottom right: number of deaths

In the United States, all states adopted restrictions related to the pandemic, with most implementing stay-at-home orders, and all closing schools and non-essential businesses. The New York Times has a website tracking the restrictions and timing of their relaxation [21]. It was broadly feared that when states reopened, cases would immediately start increasing and exponential growth would kick in. Some of the early opening states include Georgia, Missouri, Rhode Island, Colorado and Alabama, all opening the last week of April to first week of May [22]. Following are the cumulative per capita results for these states (Fig. 6).

**Fig. 7:**
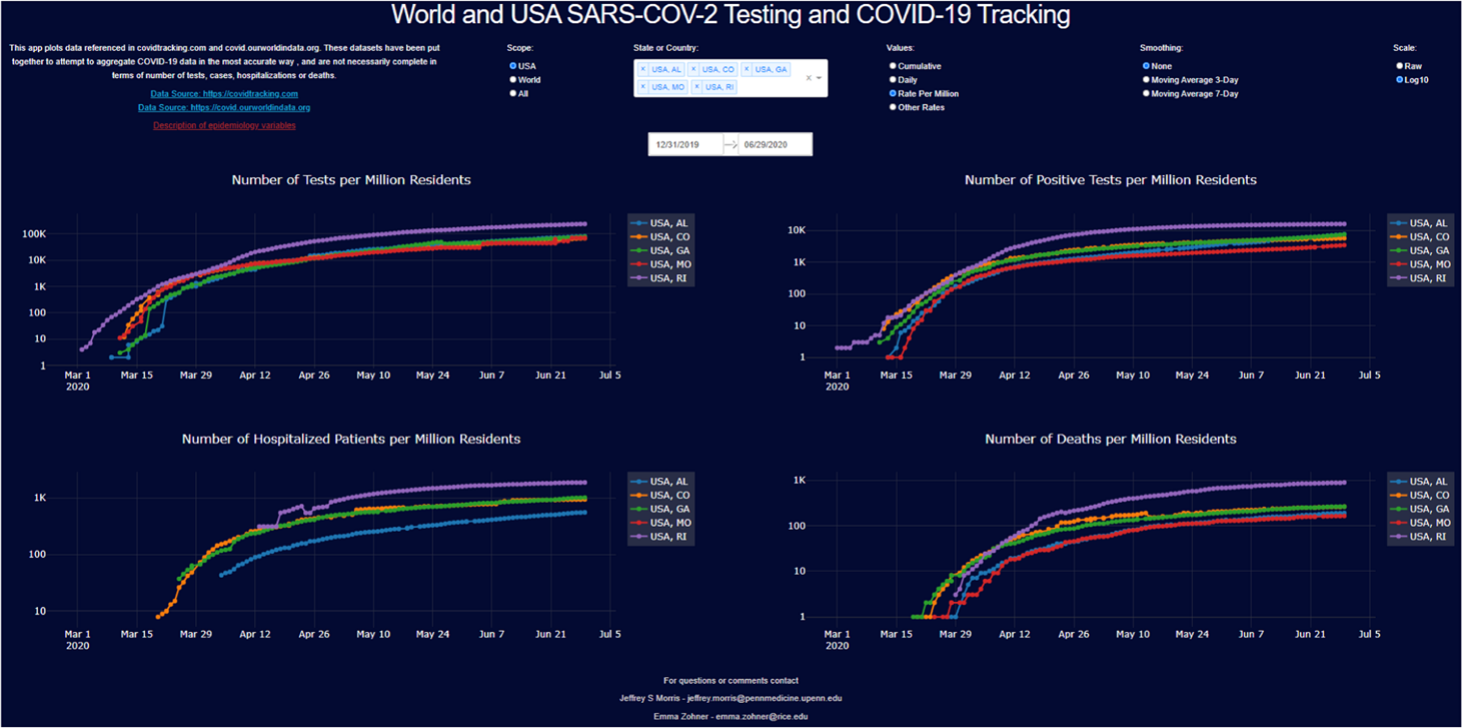
USA (AL, CO, GA, MO, RI) per million residents cumulative counts on the log scale - top left: number of tests, top right: number of cases, bottom left: number of hospitalizations, bottom right: number of deaths

We see that Rhode Island, close to the initial outbreak in New York, had higher per capita incidence than the other states, and even after opening has seen the growth of its tests, hospitalizations, and deaths continue to flatten. Colorado, Missouri, and Georgia also do not seem to have substantially increased after opening, although Alabama shows more evidence of increase. These trends are perhaps easier to see on the log scale plots (Fig. 7).

**Fig. 8:**
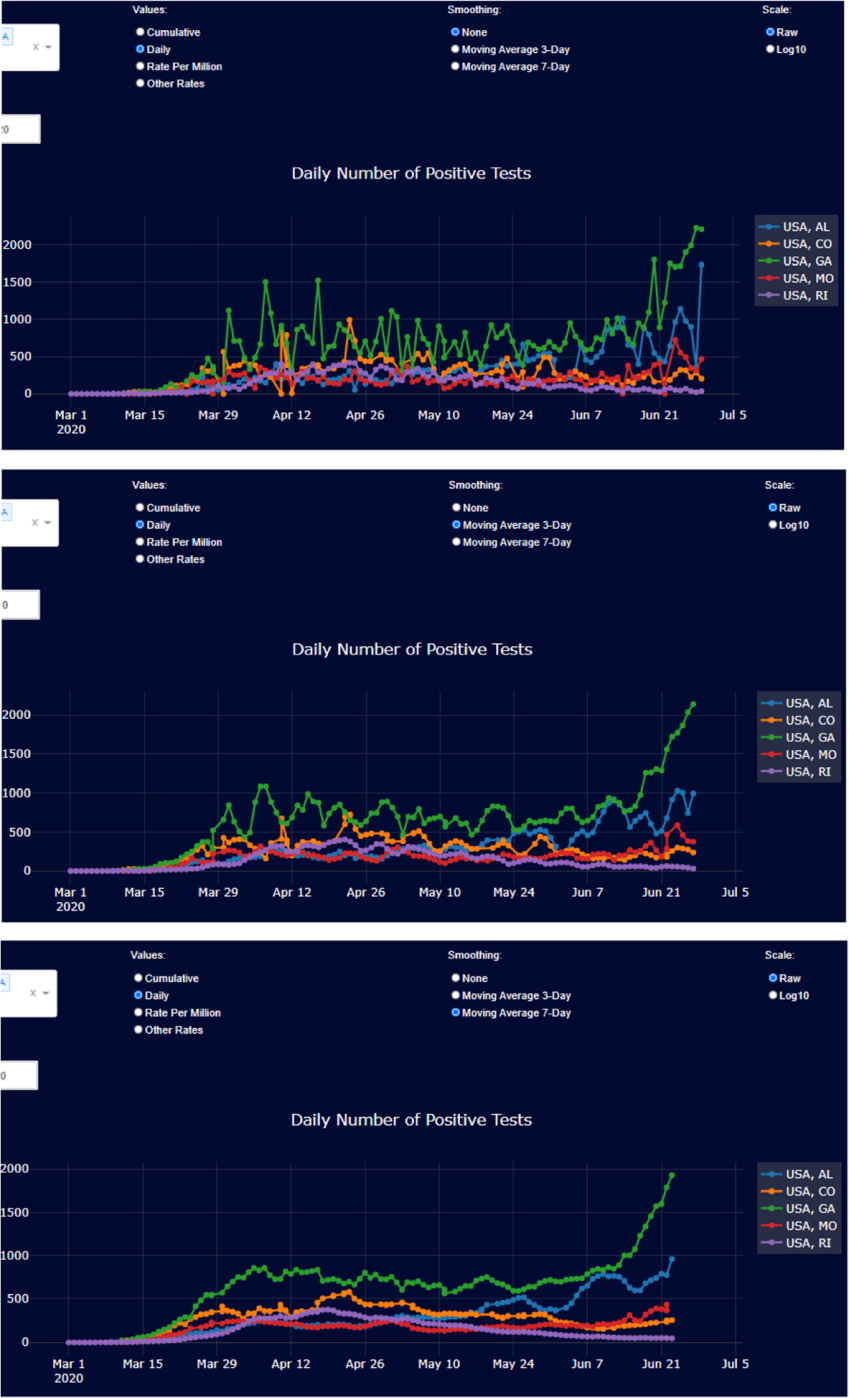
USA (AL, CO, GA, MO, RI) daily counts – no moving average, 3-day moving average and 7-day moving average

We can see the flattening of the curves more readily on the log scale. To follow the changes over time, the incremental plots showing daily counts can be more informative. However, given uneven reporting across days of the week, the daily numbers show high levels of noise that make it difficult to see the time trend. Here are the daily case counts and 3-day and 7-day moving averages, from left to right (Fig. 8).

**Fig. 9:**
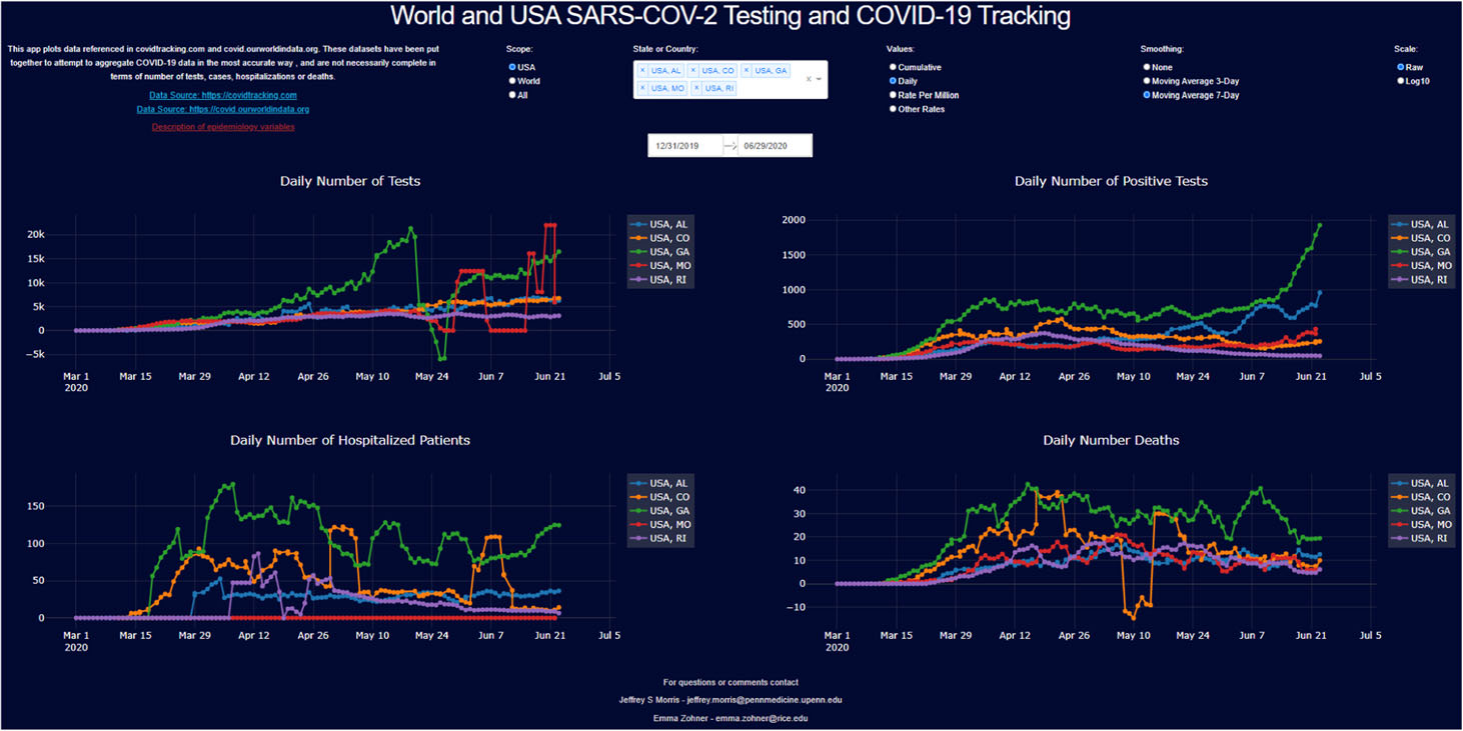
USA (AL, CO, GA, MO, RI) 7-day moving average of daily counts - top left: number of tests, top right: number of cases, bottom left: number of hospitalizations, bottom right: number of deaths

Using the 7-day moving average, we see the full panel of daily results for these states (Fig. 9 ).

We can see the continuing decrease in daily counts in Rhode Island, Colorado, and stable levels in Missouri since opening. For Georgia and Alabama, we see relatively stable values throughout the month of May, followed by a recent uptick in the last week or two in June. Overall, these states do not show any evidence of a surge or return to exponential growth, although the upticks for Georgia and Alabama in June raise some concern for a possible surge. Some other states that opened in May but were initially stable have also shown June increases that have raised concern. Let’s consider Florida, South Carolina, Texas, and Arizona, the former which opened at the beginning of May and the latter which opened at the end of May, and for comparison we will include other populous states that started opening in May, California and Pennsylvania.

### Assessing the June surge in the south and west

**Fig. 10:**
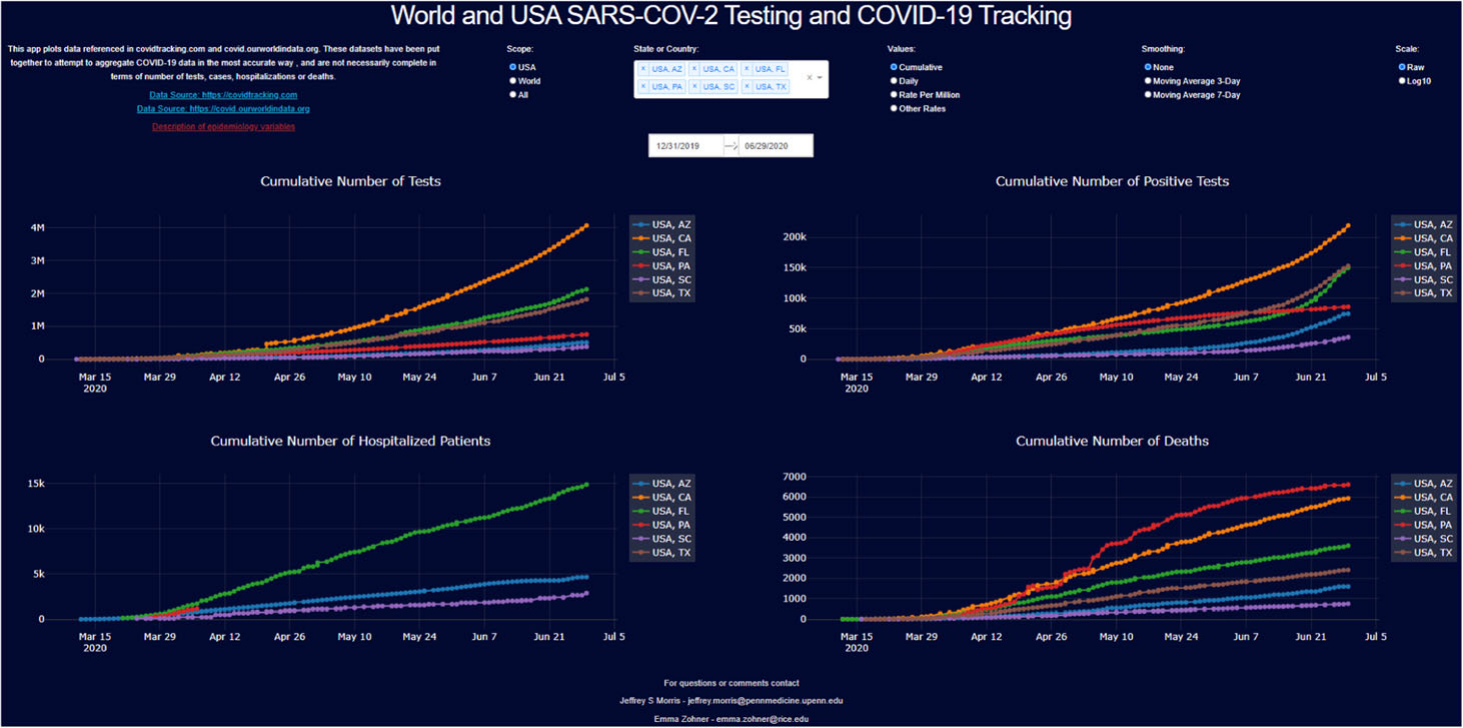
USA states (AZ, CA, FL, PA, SC, TX) cumulative counts - top left: number of tests, top right: number of cases, bottom left: number of hospitalizations, bottom right: number of deaths

Here are the cumulative counts for these state: (Fig. 10)

**Fig. 11:**
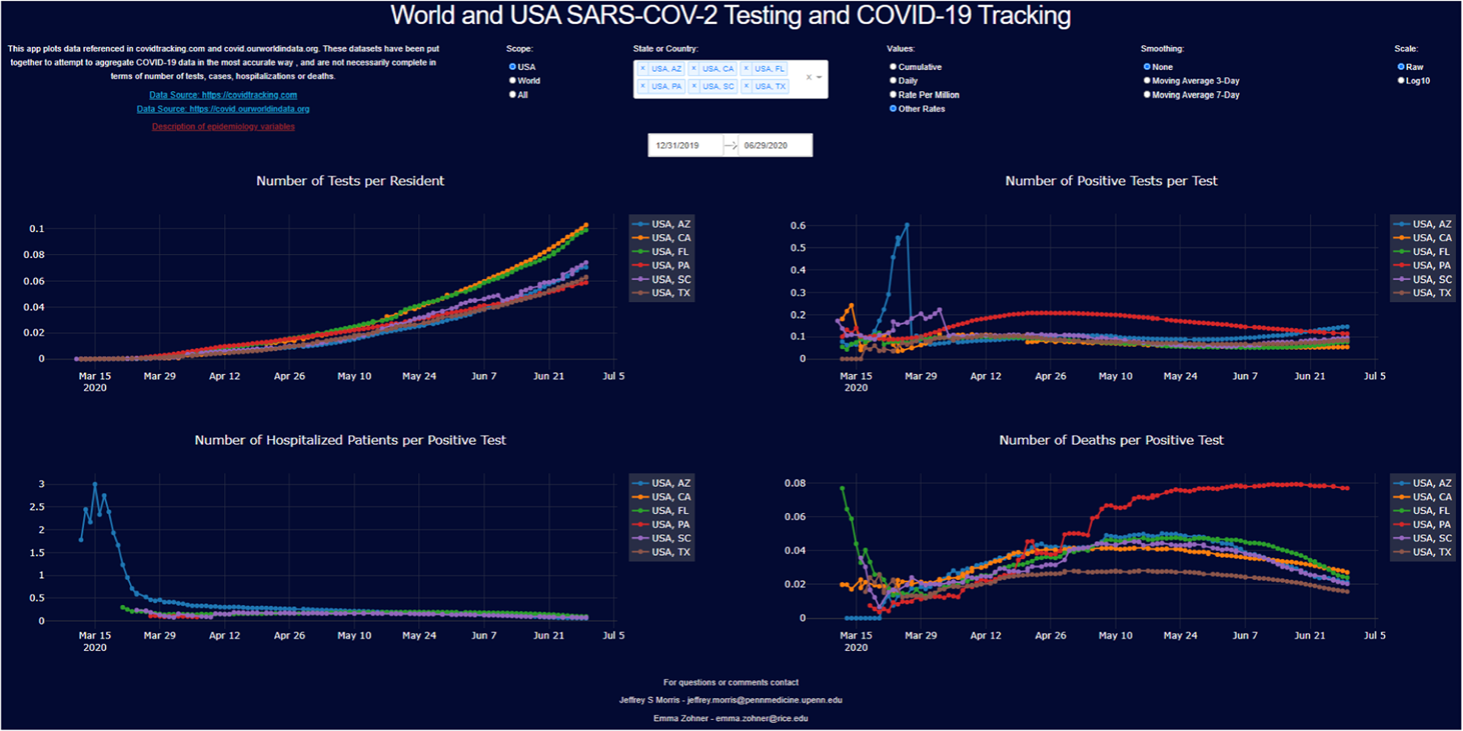
USA states (AZ, CA, FL, PA, SC, TX) rates counts - top left: number of tests per resident, top right: number of cases per test, bottom left: number of hospitalizations per positive test, bottom right: number of deaths per positive test

We see that Pennsylvania has flattened out, while California, Texas, Arizona, South Carolina, and Florida have shown increasing trends. Note that the testing rates have also climbed over time, so it is not immediately clear whether the increase in cases could be explained by increases in testing or instead is evidence of a surge in infections. Considering the test positivity rate, given under “other rates”, is helpful for assessing this. We zoomed the y-axis of this plot to focus on the region (0,0.25) to remove early artifacts from low testing rates (Fig. 11).

**Fig. 12:**
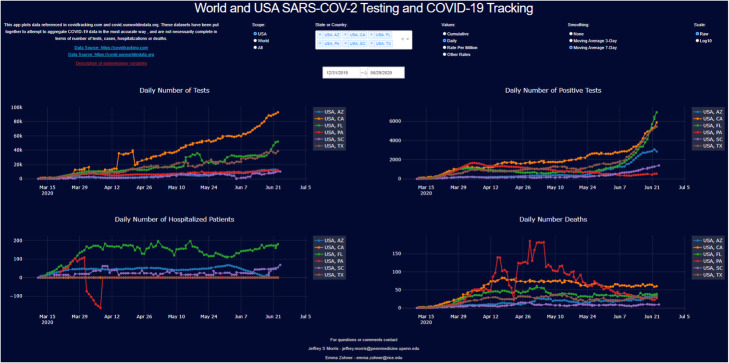
USA states (AZ, CA, FL, PA, SC, TX) 7-day moving average of daily counts - top left: number of tests, top right: number of cases, bottom left: number of hospitalizations, bottom right: number of deaths

The rate of increase in testing in California has increased as fast or slightly faster than the case count, which is reflected in decreasing test positivity rate. This suggests much of the increase in cases for California might be explainable by increased testing. This may have also been the case throughout the month of May for South Carolina, Florida, and Texas, as well, but in June we have seen an uptick in testing positivity for these states as well as Arizona. This may indicate a surge in those states. Moving averages of daily counts are useful for detecting changing points and possible surges (Fig. 12).

Here, we see that these states showed stable daily case counts that tracked closely with testing throughout the month of May, but in early June we see a surge in daily case counts for Arizona, Texas, Florida and South Carolina that is clearly of higher magnitude than any increase in testing. California’s increase in daily case counts are still closely tracked by increased testing, so may still have stable disease rates. These surges, however, show a return to exponential growth and raise concern of hospitals in these states becoming overwhelmed. The hospitalization data from covidtracking.com are spotty as data for Texas is missing and other states incomplete, so it is difficult to assess hospitalization increases for these states – local city data including Houston [23] and Phoenix [24] show hospitalizations in these cities experiencing this surge are also dramatically increasing. PA stopped entering hospitalization counts on April 6, 2020. There was a large negative entry on April 7, 2020 which causes 7 negative entries when 7-day moving average is computed. Therefore, all hospitalization counts after April 6, 2020 can be disregarded. There is not an accompanying increase in deaths yet from this surge, which may be partially due to the fact that deaths are lagged behind cases, the surge contains more young adults infected, and possibly that the virus is less virulent in this surge.

### Limitations

#### Data

The COVID Tracking Project and Our World in Data have made attempts to gather and compile the data as accurately as possible. However, because of inconsistent reporting across states and countries there are some artifacts such as negative and missing values. These are values that may be adjusted at a later date and may change values in the data.

Different jurisdictions record data differently: for example, some count a case at the date a positive test was received, which is clearly lagged since people are often not tested until experiencing symptoms, which are typically 4–10 days from infection, and there could be a week or more delay from when the sample is taken until positive tests are receive. Others, including the US state of Georgia, record a case on the date symptoms were first reported, or if unknown the day the sample was taken, or if unknown the day the positive test result was received.

The rate of reporting also varies, since some report daily while others report every few days or weekly. If data a jurisdiction does not report data on a certain date, counts are not updated until the next reported date. For example, in the case of Germany where testing data is reported weekly, one can notice a stepwise trend line rather than a smooth line such as in the case of the United States. Moving averages helps in smoothing these disparities.

Additionally, hospitalization data is not provided by most countries and many states. This measure provides a reliable, if lagged, measure of infectious spread since independent of testing, if we believe a constant proportion of infections lead to severe disease requiring hospitalization, then this should track proportionally to infection rate.

Although the stated artifacts should be noted, the compiled data and related analyses give us valuable information on characteristics of the pandemic in the United States and worldwide.

#### Application

The COVID-TRACK application provides useful visualization of the trends in COVID-19 worldwide and in the US. Some of the limitations of the web-application is that it does not support a global and a US map, which would be useful for visualizing spatial variation, and provide a convenient interface other than a drop-down list for selection of states or countries. Another limitation is that the scope is limited to countries and US states. It does not include smaller jurisdictions such as regions, counties or zip code. Improvements like this will be considered in future updated versions of the application.

## Conclusion

The COVID-19 pandemic has caused countries to take unprecedented measures to protect the health of their citizens while maintaining economic stability. The study of testing, incidence, hospitalization, and death trends can reveal invaluable insights into the emerging pandemic and the effectiveness of measures taken by different jurisdictions. The dynamic representation web-application we have developed offers a tool to scientists and others in the broader community to make comparisons at country level internationally, or at state level in the United States. The options we included in COVID-TRACK give many angles and a holistic view of available data in a clear concise manner. The insights learned from studying the data from the current pandemic will inform policy making later in the pandemic as well as during future outbreaks.

## Data Availability

The data used for COVID-TRACK are publicly available at the following two sources: The COVID Tracking Project at https://covidtracking.com for United States data, and Our World in Data at https://covid.ourworldindata.org for international data. The Python Dash code is found at https://github.com/mety19/covid-track-app/blob/master/covid_track_app.py .
